# Efficacy of a single session mindfulness based intervention: A randomized clinical trial

**DOI:** 10.1371/journal.pone.0299300

**Published:** 2024-03-13

**Authors:** Mikael Rubin, Caitlin M. Fischer, Michael J. Telch

**Affiliations:** 1 Department of Psychology, Laboratory for the Study of Anxiety Disorders, University of Texas at Austin, Austin, Texas, United States of America; 2 Palo Alto University, Palo Alto, California, United States of America; Jordan University of Science and Technology, JORDAN

## Abstract

Loneliness, perceived stress, depression, and anxiety have increased during the COVID-19 pandemic. Many of existing mindfulness and compassion-based intervention are effective, but are time-intensive, decreasing overall accessibility and scalability. Single-session interventions (SSIs) serve as a promising alternative. The current pre-registered randomized clinical trial evaluated a newly developed, manualized, mindfulness-based single-session intervention. 91 adults were randomly assigned to one of three conditions: (1) one-hour mindfulness only telehealth intervention; (b) one-hour mindfulness and compassion telehealth intervention; or (c) one-week waitlist control (before randomization to an active intervention). Intervention sessions were conducted by graduate students in clinical psychology. The primary outcome was self-reported loneliness; secondary outcomes were self-reported perceived stress, depression, and anxiety. Using Bayesian multilevel models, we found that compared to the waitlist-control, the inclusion of a compassion component led to meaningful reductions in perceived stress b = -3.75, 95% HDI [-6.95, -0.59], anxiety b = -3.79, 95% HDI [-6.99, -0.53], and depression b = -3.01, 95% HDI [-5.22, -0.78], but not loneliness at the 1-week follow-up. Results suggest that a single-session mindfulness and compassion intervention may lead to meaningful reductions in perceived stress, symptoms of anxiety, and symptoms of depression, but not loneliness. Implications of these findings are discussed.

## Introduction

COVID-19 social distancing requirements have increased rates of loneliness, an emotion linked to varied mental and physical health outcomes [1–4]. Research suggests mindfulness interventions effectively reduce feelings of loneliness [5,6]. Compassion, a component of many mindfulness practices, has also been associated with lower levels of loneliness [7,8]. While research suggests single-session interventions (SSIs) may be helpful for concerns such as depression, substance use, and anxiety, few studies have examined the efficacy of short-term interventions for loneliness [9,10].

Rates of loneliness and its associated outcomes have increased during the COVID-19 pandemic. An October 2020 national survey found among a sample of 950 adults that 36% reported experiencing severe loneliness (e.g., “frequently,” “almost all of the time,” or “all of the time”), a 9% increase from those who endorsed severe loneliness before the pandemic [11]. Furthermore, meta-analyses have found increased rates of depression (25%, a seven-fold increase compared to 3.44% in 2017) and anxiety (25%, a three-fold increase compared to 7.3% in 2017) worldwide during COVID-19 [12,13]. Czeisler et al. [14] reported two-fold increases in suicidal ideation (4.3% in 2018, 10.7% in 2020) during the COVID-19 pandemic. Moreover, one in every ten participants in their study reported starting or increasing substance use due to the COVID-19 pandemic [14]. While loneliness was not explicitly explored as a potential factor in these trends, it serves as an important therapeutic target given its known association with these outcomes. Mindfulness serves as one potential mechanism for intervening in loneliness.

Research supports the efficacy of longer-term (e.g., more than one session) mindfulness interventions for loneliness; however, they are limited in accessibility and scalability. Creswell et al. [6] found that an 8-week Mindfulness-Based Stress Reduction (MBSR) program outperformed a waitlist control in reducing loneliness among older adults. Similarly, Käll et al. [15] found that an 8-week internet-delivered Cognitive Behavioral Therapy (ICBT) treatment outperformed a waitlist control in reducing loneliness among Swedish adults. In a randomized controlled dismantling trial, Lindsay et al. [5] found that a 14-day smartphone-based mindfulness intervention reduced loneliness. While these findings are promising, these treatment protocols require an extensive time commitment, making them less accessible to individuals with time and financial constraints. The time commitment also reduces accessibility as the required time per clinician increases. Accessibility and scalability considerations become especially important when placed in the context of the widespread loneliness during the COVID-19 pandemic. A potential solution is the development of novel single-session interventions (SSIs).

SSIs have been proposed to increase accessibility and decrease financial constraints that typically accompany longer-term treatments [16–18]. Preliminary research suggests SSIs may reduce anxiety, stress and improve mental well-being in non-clinical samples [19–21]. Further, research suggests single-session mindfulness-based interventions may reduce negative affectivity (e.g., depression, rumination, anxiety, stress) [22]. Only one randomized controlled trial to date has included an assessment of the effects of a single-session intervention on loneliness [23], showing that it was effective over a six-month period compared with a control condition. Given significant elevations in loneliness during the COVID-19 pandemic [24,25], addressing loneliness with a brief intervention represents an important extension of existing SSI approaches.

Newer research suggests SSIs may be helpful for COVID-19 related distress [26]. Previous research suggests that mindfulness- and compassion-based interventions may effectively reduce feelings of loneliness [5,6,27]. However, many of these interventions are time-intensive (e.g., last multiple weeks), decreasing overall accessibility and scalability [16–18,28]. The current pre-registered clinical trial evaluated a novel mindfulness-based SSI and tested whether the incorporation of a compassion component led to more meaningful decreases in loneliness and related mental health concerns. The first hypothesis was that both mindfulness only and mindfulness + compassion interventions would lead to reductions in loneliness and perceived stress compared to a 1-week waitlist control group. The second hypothesis was that mindfulness + compassion would lead to greater reductions in loneliness at the 2-week follow-up compared to the mindfulness only group. The effects of the interventions on depression and generalized anxiety were also explored.

## Materials and methods

### Study design

This study was a 3x3 randomized controlled trial with group as a between-subjects factor with three levels (Mindfulness Only (MO), Mindfulness and Compassion (MC), and Waitlist Control (WL)) and assessment period as a three-level within-subjects factor (baseline, one-week post-treatment, and two-week follow-up). Data was collected between May 25, 2020, and November 26, 2021, via UT Qualtrics Data Capture, a HIPAA-compliant cloud-based application.

Inclusion criteria included: (1) Endorses loneliness among top three issues impacting their life; (2) Currently isolating due to COVID-19; (3) Aged 18–70 years old; (4) Fluent in English; (5) Access to the Internet with teleconferencing capabilities; (6) Access to a private setting to complete the intervention; (7) Demonstrates understanding of the constraints of the intervention; and (8) Denies suicidality. Exclusion criteria included: (1) Endorses trauma as a primary concern; (2) Endorses depression a primary concern; and (3) Has a severe mental illness (e.g., bipolar disorder, schizophrenia, borderline personality disorder).

**Fig 1:**
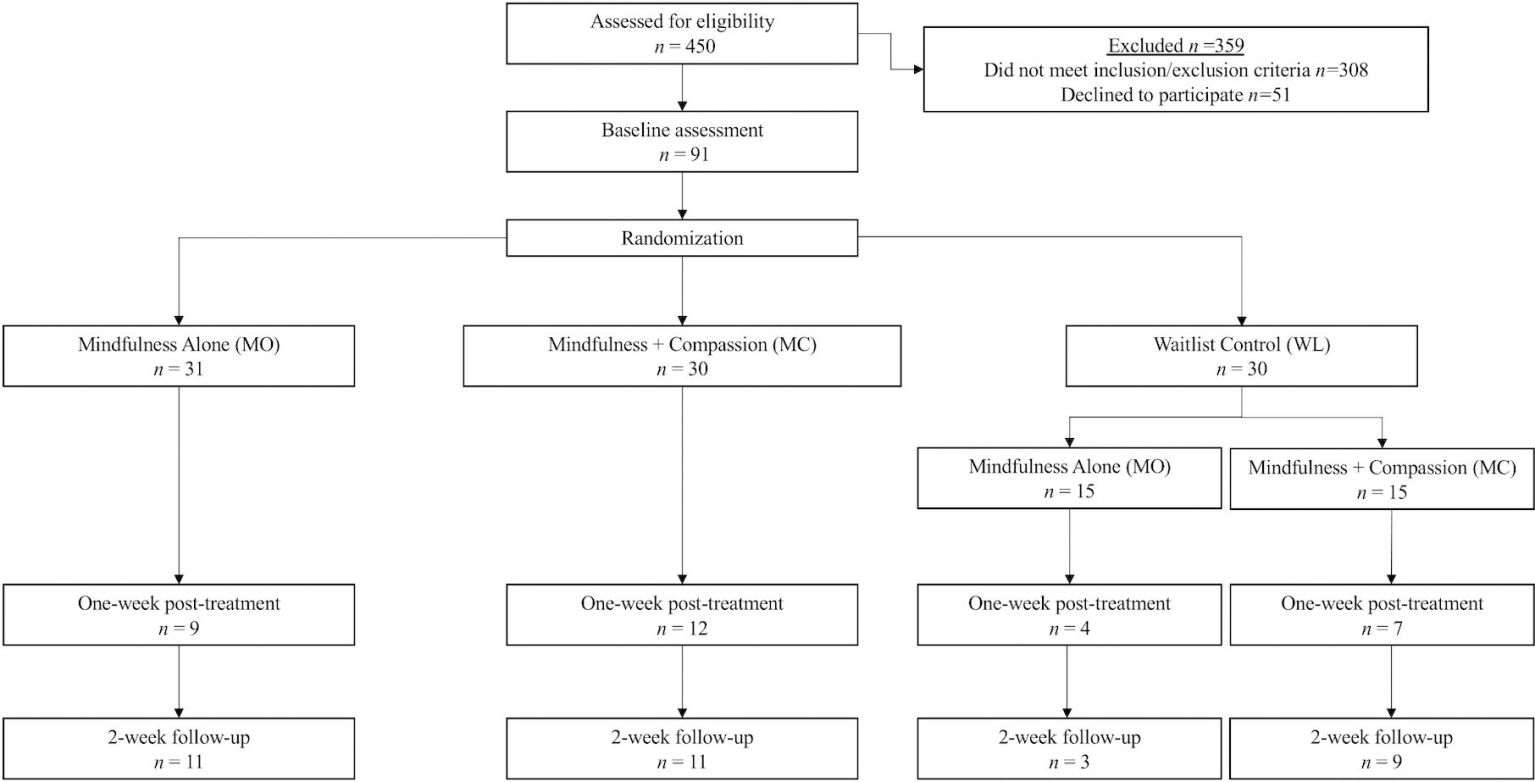
Consort flow diagram. Some participants failed to complete their one-week post-treatment assessment but completed their two-week follow-up assessment. Two participants were excluded due to not meeting eligibility criteria (n = 1, MO; n = 1, WL MC).

Participants provided written informed consent via Qualtrics. After signing the informed consent document, participants were randomized to one of the three conditions: (1) one-hour mindfulness only telehealth intervention (MO); (b) one-hour mindfulness and compassion telehealth intervention (MC); and (c) one-week waitlist control (before randomization to an active intervention; WL). The one-hour intervention followed the Single Session Mindfulness Intervention for Loneliness (SSMILe) protocol which is available at the following link: https://doi.org/10.17605/OSF.IO/9DTNS [29]. The compassion module was reserved to be completed only by those in the mindfulness and compassion condition. The study flow is detailed in Fig 1. Specific components of this intervention are outlined below. All study procedures were approved by the University of Texas at Austin IRB, NCT04414826.

#### Randomization

We utilized block randomization with a block-size of 6. Block randomization was conducted in R by the first author using the *blockrand* package [30]. This code is available. Participants were enrolled and randomized by the second author. Allocation was concealed from the primary clinician of the study. The study was single-blinded (participants were theoretically blind to the intervention condition although it would be possible to deduce, but the clinician could not be blinded due to the nature of the interventions).

#### Sample size estimation

We conducted a simulation study to test the power to confidently reject the null across a range of sample sizes per group (10, 15, 20, and 30). We conducted 100 simulations of each model for the primary outcome variable and evaluated power by determining the proportion of times we rejected the null using an 89% Highest Density Interval (HDI) with a custom Region of Practical Equivalence (ROPE) interval using the rope_range function. 30 participants per group provided > 90% power for each planned test. The planned sample size was intended to accommodate a reasonable degree of attrition, but was not specifically tested in the power analysis. The R syntax used for the simulations is available.

### Intervention components

The intervention consisted of a single one-hour-long telehealth session conducted by a doctoral student in clinical psychology via a HIPAA-complaint teleconferencing platform. Participants were sent an email the morning of their scheduled intervention appointment, including the appointment time and hyperlink (using HIPAA-compliant Zoom). Immediately after the intervention appointment, participants were provided a survey link where they were asked to provide feedback on their appointment. At this time, participants were also provided with a handout discussing specific topics and skills covered during the intervention.

During the intervention appointment, the taught skills were presented as a hierarchy, building off one another and capable of being practiced independently. At the end of each skill module, time was provided to discuss participant takeaways and answer questions. Participants were provided the option to turn their video and audio off for the duration of each exercise.

#### Rapport-building and psychoeducation

The first ten minutes of the intervention (approximately) involved psychoeducation on the constructs of loneliness and mindfulness. Study clinicians used participants’ own examples to individualize the intervention and build rapport. Participants were first provided information on the differences between “being alone” and feeling lonely. Consistent with previous literature, the therapists contextualized loneliness as an uncomfortable emotion that often results in engagement in ineffective coping behaviors (e.g., avoidance) [31,32]. Then, mindfulness was introduced as an effective tool for coping with loneliness by lessening the subjective distress attached to this emotion [33,34].

#### Skill practice: Awareness

Awareness was presented as a tool for centering one’s attention on the present moment [34]. This skill was practiced via belly breathing, where each participant was instructed to focus on the sensations of breathing in their stomach for two to three minutes. Participants were instructed to note any sensations and to refocus their attention if they found their mind wandering.

#### Skill practice: Non-reactivity and non-judgment

The second mindfulness skill taught centered around two facets of acceptance: non-judgment of and non-reactivity to inner experiences [35]. This skill module was presented as a method for sitting with the discomfort associated with loneliness and creating “space” between this discomfort and reflexive reactions [32,34]. Participants were first prompted to scan their bodies for uncomfortable thoughts or physiological sensations associated with their subjective experience of loneliness. If feelings of loneliness were not present, participants were asked to scan their bodies for other uncomfortable sensations. Participants were given the option to identify an uncomfortable thought if they could not identify any uncomfortable sensations.

Once participants identified an uncomfortable sensation or thought, they were instructed to focus on it in a non-judgmental, curious manner. Again, an emphasis was placed on refocusing attention if they found their minds wandering. Last, participants were informed they could return their awareness to their breath if focusing on their chosen uncomfortable thought or sensation became too overwhelming. Participants were encouraged to make this switch with a non-judgmental attitude. Participants practiced this exercise for approximately two to three minutes.

#### Skill practice: Compassion

Those assigned to the mindfulness and compassion intervention were taught a third skill related to compassion. First, compassion, or feelings of warmth and care, was differentiated from empathy or sharing others’ feelings [36]. This skill module was presented as a method for becoming familiar with what compassion physiologically feels like to recognize one’s underlying behavioral motivations better. Participants were instructed to think of a person, place, object, or spiritual or religious figure that consistently evokes feelings of warmth, love, kindness, or whatever compassion feels like to them [29]. They were encouraged to focus their attention on any sensations that arose after evoking this feeling. Like previous exercises, they were instructed to notice and refocus their attention if their mind wandered. Participants were encouraged to re-evoke feelings of compassion if they dissipated. This skill was practiced for two to three minutes.

### Measures

#### Primary outcome measure

*Revised UCLA Loneliness Scale-8* (ULS-8) [37]. The ULS-8 is an 8-item self-report measure that assesses subjective feelings of loneliness and social isolation. Participants were asked to rate how often they experience each item on a 4-point scale from “never” to “often.” Individual item scores were summed to provide an overall loneliness score, with higher scores indexing greater feelings of loneliness. The ULS-8 is highly correlated with the ULS-20 (r = 0.91) and demonstrated adequate internal consistency in the current sample (Cronbach’s alpha = 0.76) [37].

#### Secondary outcome measure

*Perceived Stress Scale* (PSS) [38]. The PSS is a 10-item scale that assesses subjective feelings of stress. Participants were asked to rate how often they felt or thought each item on a 5-point scale from “never” to “very often” in the past week. Individual item scores were summed to provide an overall score. The 10-item PSS demonstrated adequate internal consistency in the current sample (Cronbach’s alpha = 0.76).

#### Exploratory outcome measures

*Patient Health Questionnaire– 8* (PHQ-8) [39]. The PHQ-8 is an 8-item scale assessing the severity of depressive symptoms. Participants rated how often they were bothered by each problem on a 4-point scale from “not at all” to “nearly every day” in the past week. Participants who endorsed a score of 15 or greater on this measure (e.g., “moderately severe”) and who endorsed these symptoms as a primary concern were excluded from participation [39]. The PHQ-8 has demonstrated strong construct validity, specificity, and sensitivity [39]. This measure demonstrated adequate internal consistency in this sample (Cronbach’s alpha = 0.76).

*Generalized Anxiety Disorder– 7* (GAD-7) [40]. The GAD-7 is a 7-item self-report measure that assesses the severity of anxiety symptoms. Participants were asked to rate how often they have been bothered by each symptom on a 4-point scale from “not at all” to “nearly every day” in the past week. The total score was taken by summing individual item scores. Higher scores represent greater severity of anxiety symptoms. This measure demonstrated adequate internal consistency in this sample (Cronbach’s alpha = 0.85).

Treatment Validity Measure. *Credibility/Expectancy Questionnaire* (CEQ) [41]. The CEQ is a 6-item measure that examines how credible (i.e., convincing and logical) the individual thinks treatment is and how much they believe they will experience improvements because of treatment (“expectancy”) [41,42]. The credibility subscale (comprised of three items) is rated on a 9-point scale from “not at all logical” to “very logical”. Expectancy subscale items are rated from 0 to 100% in ten percentage point increments. This questionnaire demonstrated adequate internal consistency in this sample (Cronbach’s alpha = 0.87).

### Data analysis

#### Interim analysis

An interim analysis (n = 86) was conducted and reported in a master’s thesis [unpublished]. The results of this interim analysis are consistent with those of the present analysis.

#### Present analyses

All analyses were completed in R version 4.1.2, using the *brms* package [43]. We conducted Bayesian multilevel models to address the primary outcomes, with random intercepts for each individual. All participants who completed the intervention were included in the analyses. Missing data was imputed using one-step multiple imputation with the integrated solution provided by *brms* as detailed below. To address the pre-registered hypotheses, we tested the effects of active treatment conditions against the waitlist-control at the 1-week follow-up for self-reported loneliness and stress as well as compared self-reported loneliness and stress between the active conditions at the 2-week follow-up. Exploratory pre-registered analyses replicated this analytic approach (active conditions compared against waitlist control at 1-week and between active conditions at 2-weeks) for self-reported symptoms of anxiety and depression. Pre-registered hypotheses were tested using weakly informative priors as well as uninformative priors (centered on zero). There were several deviations from the pre-registered approach: (1) rather than use 89% intervals, we use 95% intervals which is more standard; (2) we did not use a ‘rope_range’ function given the use of the narrower confidence interval; (3) multiple imputation was conducted within the models (using *mi()* specification which uses the model and priors to generate a distribution of plausible values for each missing data point) instead of external imputation with the *mice* package because it more readily takes into account the priors and multilevel nature of the data and the imputation accuracy can be examined using posterior predictive checks. We report Bayes Factors (BFs) computed using the *hypothesis* function. For models with weakly informative priors, we tested the bias of the parameter estimates by comparing them to models with uninformative priors. All syntax and data needed to reproduce the results reported is available at https://osf.io/bd4t3.

## Results

### Demographics

This study’s sample comprised 91 adult participants. The mean age for the sample was 27.32 years. Approximately 60.44% of the sample was female, 61.54% was White or Euro-American, and 76.92% was not Hispanic or Latino. The majority (76.92%) of the sample was recruited from the community (the remainder from the undergraduate research pool at the University of Texas at Austin). Demographic information can be found in Table 1.

**Table 1:** Participant demographics.

	Mindfulness Only (n = 31)	Mindfulness and Compassion (n = 30)	Waitlist (n = 30)
	M (SD)	M (SD)	M (SD)
Age	25.79 (7.99)	28.63 (12.42)	27.48 (11.72)
CEQ Credibility CEQ Expectancy	6.92 (1.22) 24.28 (10.66)	6.85 (1.03) 32.10 (11.98)	6.50 (0.83) 27.95 (15.76)
GAD-7 PSS PHQ-8 ULS-8	8.14 (4.37) 22.55 (4.04) 9.38 (3.79) 23.69 (4.69)	8.10 (5.43) 20.67 (3.89) 10.30 (4.75) 23.63 (4.33)	7.28 (4.54) 21.79 (4.58) 8.07 (4.67) 23.00 (4.39)
	N (%)	N (%)	N (%)
Female Prefer not to answer	16 (52%) 3 (10)	20 (67%) 0	19 (63%) 1 (3)
Hispanic/Latino Prefer not to answer	6 (19) 3 (10)	3 (10) 1 (3)	7 (23) 1 (3)
Race			
American Indian or Alaska Native	1 (3)	0	0
Asian	4 (13)	4 (13)	9 (30)
Black or African American	2 (7)	3 (10)	2 (7)
White Other Prefer not to answer	18 (58) 2 (7) 4 (13)	22 (73) 1 (3) 0	16 (53) 2 (7) 1 (3)
Recruited from community	23 (74)	26 (87)	21 (70)

CEQ = Credibility/Expectancy Questionnaire. GAD-7 = Generalized Anxiety Disorder– 7. PSS = Perceived Stress Scale. PHQ-8 = Patient Health Questionnaire– 8. ULS-8 = Revised UCLA Loneliness Scale-8.

### Primary outcome

There was evidence suggesting that neither the mindfulness + compassion (MC) *b* = -1.60, 95% Highest Density Interval (HDI) [-3.52, 0.33], Bayes Factor (BF) = 0.67 nor mindfulness only (MO) *b* = -0.78, 95% HDI [-2.82, 1.24], BF = 0.24 interventions decreased loneliness meaningfully compared to the waitlist control condition at the 1-week follow-up.

Comparing the two intervention conditions, there was evidence against a meaningful difference at the 1-week follow-up *b* = -0.88, 95% HDI [-2.88, 1.12], BF = 0.26 or the 2-week follow-up *b* = 0.56, 95% HDI [-1.42, 2.52], BF = 0.21. There was also no meaningful main effect of time at the 1-week follow-up *b* = -0.82, 95% HDI [-1.85, 0.20], BF = 0.31; however, at 2-week follow-up there was a meaningful main effect of time *b* = -2.36, 95% HDI [-3.36, -1.36], BF > 1,000. All results reported used multiple imputation as described in the methods section.

### Secondary outcome

At the 1-week follow-up for perceived stress there was an effect of mindfulness + compassion *b* = -3.75, 95% HDI [-6.95, -0.59], BF = 3.63, but not for mindfulness only *b* = -1.10, 95% HDI [-4.47, 2.27], BF = 0.37, compared to the waitlist condition.

There were no meaningful differences between the intervention conditions at the 1-week *b* = -2.09, 95% HDI [-5.56, 1.47], BF = 0.74 or 2-week *b* = 1.23, 95% HDI [-2.23, 4.67], BF = 0.44 follow-ups. There was also no meaningful main effect of time at the 1-week follow-up across conditions *b* = -1.23, 95% HDI [-3.17, 0.70], BF = 0.43; however, at 2-week follow-up there was a meaningful main effect of time *b* = -2.22, 95% HDI [-4.15, -0.29], BF = 2.44. All results reported used multiple imputation as described in the methods section.

### Exploratory outcomes

There was a meaningful effect of mindfulness + compassion compared to waitlist on symptoms of depression *b* = -3.01, 95% HDI [-5.22, -0.78], BF = 7.14 and anxiety *b* = -3.79, 95% HDI [-6.99, -0.53], BF = 4.83 at the 1-week follow-up. There was no meaningful effect of mindfulness only compared to waitlist on symptoms of depression *b* = -0.59, 95% HDI [-2.89, 1.76], BF = 0.26 or anxiety *b* = -1.53, 95% HDI [-4.79, 1.78], BF = 0.51 at the 1-week follow-up.

There were no meaningful differences between mindfulness + compassion compared to mindfulness alone at the 1-week follow-up for symptoms of depression *b* = -1.08, 95% HDI [-3.83, 1.70], BF = 0.38 or anxiety *b* = -1.50, 95% HDI [-4.39, 1.45], BF = 0.51 or at the 2-week follow-up for symptoms of depression *b* = 0.33, 95% HDI [-2.46, 2.97], BF = 0.39 or anxiety *b* = -1.54, 95% HDI [-4.38, 1.34], BF = 0.52. Table 2 highlights mean scores on each outcome variable by group and time point. Tables 3 and 4 summarize the results of our two primary comparisons: (1) waitlist condition versus active conditions (Table 3); and (2) mindfulness + compassion versus mindfulness alone (Table 4). All results reported used multiple imputation as described in the methods section.

**Table 2:** Outcome variables across groups by assessment period.

	Baseline			1-week Follow-Up			2-week Follow-Up	
	MO (n = 31)	MC (n = 30)	WL (n = 30)	MO (n = 9)	MC (n = 12)	WL (n = 22)	MO (n = 14)	MC (n = 20)
ULS-8	23.69 (4.69)	23.63 (4.33)	23.00 (4.39)	24.70 (3.97)	21.10 (3.06)	23.09 (3.74)	21.90 (3.36)	21.30 (4.28)
PSS	22.55 (4.04)	20.67 (3.89)	21.79 (4.58)	24.90 (6.29)	18.20 (7.06)	21.86 (3.58)	19.20 (5.09)	20.20 (3.25)
GAD-7	8.14 (4.37)	8.10 (5.43)	7.28 (4.54)	9.67 (5.05)	5.33 (2.74)	9.81 (4.86)	8.71 (4.21)	5.50 (2.98)
PHQ-8	9.38 (3.79)	10.30 (4.75)	8.07 (4.67)	9.89 (6.66)	5.92 (3.23)	8.09 (5.20)	7.71 (3.38)	6.85 (4.77)

Values are presented as Mean (Standard Deviation). ULS-8 = Revised UCLA Loneliness Scale-8. PSS = Perceived Stress Scale. GAD-7 = Generalized Anxiety Disorder– 7. PHQ-8 = Patient Health Questionnaire– 8.

**Table 3:** Waitlist versus active condition outcome analyses.

	ULS-8				PSS	PHQ-8		GAD-7	
*Predictors*	*Estimates*	*CI (95%)*		*Estimates*	*CI (95%)*	*Estimates*	*CI (95%)*	*Estimates*	*CI (95%)*
Intercept	24.06	22.53–25.58		21.71	19.33 – 24.13	7.89	5.98–9.83	7.49	5.72–9.24
WLMC	-1.73	-4.09–0.65		-1.45	-5.07–2.10	-0.35	-3.24–2.55	-1.54	-4.08–1.04
WLMO	1.01	-1.27–3.28		2.13	-1.53–5.63	1.58	-1.22–4.44	1.01	-1.53–3.59
t: t1	-0.67	-1.52–0.18		-0.00	-1.50–1.54	-0.31	-1.32–0.72	1.18	-0.35–2.71
WLMC:t1	-1.60	-3.52–0.33		-3.75	-6.95 – -0.59	-3.01	-5.22 – -0.78	-3.79	-6.99 – -0.53
WLMO:t1	-0.78	-2.82–1.24		-1.10	-4.47 – 2.27	-0.59	-2.89 – 1.76	-1.53	-4.79–1.78
**Random Effects**									
σ^2^	3.61			11.37		4.83		11.20	
τ_00_	10.74 _id_			26.64 _id_		16.48 _id_		8.70 _id_	
ICC	0.75			0.70		0.77		0.44	
N	50 _id_			50 _id_		50 _id_		50 _id_	

ULS-8 = Revised UCLA Loneliness Scale-8. PSS = Perceived Stress Scale. PHQ-8 = Patient Health Questionnaire– 8. GAD-7 = Generalized Anxiety Disorder– 7.

**Table 4:** Active condition (mindfulness + compassion versus mindfulness alone) outcome analyses.

	ULS-8		PSS		PHQ-8		GAD-7	
*Predictors*	*Estimates*	*CI (95%)*	*Estimates*	*CI (95%)*	*Estimates*	*CI (95%)*	*Estimates*	*CI (95%)*
Intercept	24.25	22.73–25.77	23.14	20.96–25.30	9.55	7.96–11.14	8.59	7.07–10.11
MC	-0.64	- 2.71–1.44	- 2.38	- 5.19–0.38	- 2.47	- 4.52 - -0.41	- 2.41	- 4.25 - -0.53
t: t1	-0.82	-1.85–0.20	- 1.23	- 3.17–0.70	- 1.01	- 2.46–0.45	- 0.53	- 2.07–1.04
t: t2	-2.36	- 3.36 - -1.36	-2.22	- 4.15 - - 0.29	- 1.29	- 2.71–0.12	- 0.53	- 2.05–1.01
MC:t1	-0.88	- 2.88–1.12	-2.09	-5.56–1.47	- 1.08	- 3.83–1.70	- 1.50	- 4.39–1.45
MC:t2	0.56	- 1.42–2.52	1.23	- 2.23–4.67	0.33	- 2.46–2.97	- 1.54	- 4.38–1.34
**Random Effects**								
σ^2^	4.44		16.74		9.41		11.05	
τ_00_	11.64 _id_		18.45 _id_		9.14 _id_		5.91 _id_	
ICC	0.72		0.52		0.49		0.35	
N	50 _id_		50 _id_		50 _id_		50 _id_	

## Discussion

This was the first single session mindfulness intervention intended to specifically target symptoms of loneliness. Given the degree to which the COVID-19 pandemic has led to increases in loneliness and stress, it was important to consider ways to address this concern in a relatively accessible and brief way. Contrary to our expectations, there was no meaningful effect for either intervention on loneliness compared to waitlist at the 1-week follow-up and we did not find any group differences between the active intervention conditions at the 1-week or 2-week follow-ups. However, we did find that by the 2-week follow-up there was a moderate decrease in loneliness across both conditions. Without a control condition, however, further research is needed to determine whether this change is truly an effect of the intervention or related to natural attenuation of loneliness over time.

Our secondary and exploratory analyses indicated that at the 1-week follow-up the mindfulness + compassion condition, but not the mindfulness only condition, had meaningful reductions across perceived stress, symptoms of depression and anxiety compared to the waitlist condition. It is worth noting that we did not find any meaningful differences between the two mindfulness conditions at the 1-week or 2-week follow-ups. Taken together, these findings suggest that a single session (1 hour) mindfulness intervention with a compassion component may be effective for reducing perceived stress, symptoms of anxiety, and symptoms of depression compared to no intervention. The effect sizes in the current study are somewhat smaller than in previous research which also had much longer intervention protocols (e.g., 8 sessions) and this difference is in line with comparisons between other single session interventions (e.g., for depression) and longer treatment protocols. We did not find meaningful differences between the mindfulness conditions, however. It may be that incorporating a compassion component leads to modest reductions in symptoms in the short-term. The lack of meaningful differences between the active intervention conditions may reflect the brevity of the compassion component (participants spent only 5–10 minutes learning about and practicing compassion) and the lack of longer follow-up assessments. Future research might test a longer compassion component to determine whether greater attention to this aspect in the context of a brief intervention for loneliness and stress is more helpful.

The present study had several limitations that are important to note. First, the sample size was small. We attempted to minimize this through the use of Bayesian analysis, which is well-equipped to handle data from small samples. Second, the follow-up period was short. Longer follow-up may provide better insight into the efficacy of the mindfulness interventions on loneliness as well as the role of compassion compared to mindfulness alone. It is possible that our short follow-up periods failed to capture delayed effects of our intervention. Third, the sample was non-clinical and most participants reported mild-moderate symptoms of anxiety and depression. Additionally, in some respects our sample was not diverse, with every participant requiring access to the Internet with videoconferencing capabilities and the majority of participants identifying as White. Lastly, the loneliness measure did not provide a symptom time window. This may have led to heterogeneous interpretation of when the loneliness symptoms were in reference to; given the relatively short follow-up duration this is an important limitation. Consequently, our results should be interpreted with caution. Future research with larger and more diverse samples may enhance generalizability of our findings.

These findings suggest that a single session mindfulness intervention can lead to meaningful reductions across a range of clinical concerns, including perceived stress, anxiety, and depressive symptoms. This brief single session mindfulness intervention offers an approach that can be easily adopted in a range of contexts. It is important for future research to evaluate this approach with larger samples and to examine whether changes in symptoms are maintained over longer periods of time.

## Supporting information

S1 File (PDF)

S2 File (PDF)

## References

[1] Shankar A, McMunn A, Banks J, Steptoe A. Loneliness, social isolation, and behavioral and biological health indicators in older adults. Health Psychol [Internet]. 2011 Jul;30(4):377–85. Available from: doi: 10.1037/a0022826 21534675

[2] Cacioppo JT, Hughes ME, Waite LJ, Hawkley LC, Thisted RA. Loneliness as a specific risk factor for depressive symptoms: cross-sectional and longitudinal analyses. Psychol Aging [Internet]. 2006 Mar;21(1):140–51. Available from: doi: 10.1037/0882-7974.21.1.140 16594799

[3] Thamboo PA. The Effects of a Mindfulness-Based Intervention on Feelings of Loneliness and Ruminative Thinking. Available from: http://digitalcommons.brockport.edu/psh_theses.

[4] Rich AR, Kirkpatrick-Smith J, Bonner RL, Jans F. Gender differences in the psychosocial correlates of suicidal ideation among adolescents. Suicide Life Threat Behav [Internet]. 1992 Autumn;22(3):364–73. Available from: https://www.ncbi.nlm.nih.gov/pubmed/1440750. 1440750

[5] Lindsay EK, Young S, Brown KW, Smyth JM, Creswell JD. Mindfulness training reduces loneliness and increases social contact in a randomized controlled trial. Proc Natl Acad Sci U S A [Internet]. 2019 Feb 26;116(9):3488–93. Available from: doi: 10.1073/pnas.1813588116 30808743 PMC6397548

[6] Creswell JD, Irwin MR, Burklund LJ, Lieberman MD, Arevalo JMG, Ma J, et al. Mindfulness-Based Stress Reduction training reduces loneliness and pro-inflammatory gene expression in older adults: a small randomized controlled trial. Brain Behav Immun [Internet]. 2012 Oct;26(7):1095–101. Available from: doi: 10.1016/j.bbi.2012.07.006 22820409 PMC3635809

[7] Lyon TA. Self-compassion as a predictor of loneliness: the relationship between self-evaluation processes and perceptions of social connection. 2015; Available from: http://firescholars.seu.edu/cgi/viewcontent.cgi?article=1038&context=honors.

[8] Akin A. Self-compassion and Loneliness. International Online Journal of Educational Sciences [Internet]. 2010;2(3). Available from: http://citeseerx.ist.psu.edu/viewdoc/download?doi=10.1.1.422.382&rep=rep1&type=pdf.

[9] Maljanen T, Knekt P, Lindfors O, Virtala E, Tillman P, Härkänen T, et al. The cost-effectiveness of short-term and long-term psychotherapy in the treatment of depressive and anxiety disorders during a 5-year follow-up. J Affect Disord [Internet]. 2016 Jan 15;190:254–63. Available from: doi: 10.1016/j.jad.2015.09.065 26540079

[10] Jarrett RB, Rush AJ. Short-term psychotherapy of depressive disorders: current status and future directions. Psychiatry [Internet]. 1994 May;57(2):115–32. Available from: doi: 10.1080/00332747.1994.11024675 7938331

[11] Weissbourd R, Batanova M, Lovison V, Torres E. Loneliness in America how the Pandemic Has Deepened an Epidemic of Loneliness and What We Can Do about it. Harvard University. https://static1. squarespace. com/static …; 2021.

[12] Bueno-Notivol J, Gracia-García P, Olaya B, Lasheras I, López-Antón R, Santabárbara J. Prevalence of depression during the COVID-19 outbreak: A meta-analysis of community-based studies [Internet]. Vol. 21, International Journal of Clinical and Health Psychology. 2021. p. 100196. Available from: doi: 10.1016/j.ijchp.2020.07.007 32904715 PMC7458054

[13] Santabárbara J, Lasheras I, Lipnicki DM, Bueno-Notivol J, Pérez-Moreno M, López-Antón R, et al. Prevalence of anxiety in the COVID-19 pandemic: An updated meta-analysis of community-based studies. Prog Neuropsychopharmacol Biol Psychiatry [Internet]. 2021 Jul 13;109:110207. Available from: doi: 10.1016/j.pnpbp.2020.110207 33338558 PMC7834650

[14] Czeisler MÉ, Lane RI, Petrosky E, Wiley JF, Christensen A, Njai R, et al. Mental Health, Substance Use, and Suicidal Ideation During the COVID-19 Pandemic—United States, June 24–30, 2020. MMWR Morb Mortal Wkly Rep [Internet]. 2020 Aug 14;69(32):1049–57. Available from: doi: 10.15585/mmwr.mm6932a1 32790653 PMC7440121

[15] Käll A, Jägholm S, Hesser H, Andersson F, Mathaldi A, Norkvist BT, et al. Internet-Based Cognitive Behavior Therapy for Loneliness: A Pilot Randomized Controlled Trial. Behav Ther [Internet]. 2020 Jan;51(1):54–68. Available from: doi: 10.1016/j.beth.2019.05.001 32005340

[16] Campbell A. Single-session approaches to therapy: Time to review. Aust N Z J Fam Ther [Internet]. 2012 Mar;33(01):15–26. Available from: http://doi.wiley.com/10.1017/aft.2012.3.

[17] Bobele M, López SS-G, Scamardo M, Solórzano B. Single-Session/Walk-In Therapy with Mexican-American Clients. Journal of Systemic Therapies [Internet]. 2008 Dec 1;27(4):75–89. Available from: doi: 10.1521/jsyt.2008.27.4.75

[18] Schleider JL, Weisz JR. Little treatments, promising effects? Meta-analysis of single-session interventions for youth psychiatric problems. J Am Acad Child Adolesc Psychiatry [Internet]. 2017 Feb;56(2):107–15. Available from: https://linkinghub.elsevier.com/retrieve/pii/S0890856716319335. 28117056 10.1016/j.jaac.2016.11.007

[19] Keogh E, Bond FW, Flaxman PE. Improving academic performance and mental health through a stress management intervention: outcomes and mediators of change. Behav Res Ther [Internet]. 2006 Mar;44(3):339–57. Available from: doi: 10.1016/j.brat.2005.03.002 15890314

[20] Finkelstein C, Brownstein A, Scott C, Lan Y-L. Anxiety and stress reduction in medical education: an intervention. Med Educ [Internet]. 2007 Mar;41(3):258–64. Available from: doi: 10.1111/j.1365-2929.2007.02685.x 17316210

[21] Häfner A, Stock A, Oberst V. Decreasing students’ stress through time management training: an intervention study. European Journal of Psychology of Education [Internet]. 2015 Mar 1;30(1):81–94. Available from: doi: 10.1007/s10212-014-0229-2

[22] Schumer MC, Lindsay EK, Creswell JD. Brief mindfulness training for negative affectivity: A systematic review and meta-analysis. J Consult Clin Psychol [Internet]. 2018 Jul;86(7):569–83. Available from: doi: 10.1037/ccp0000324 29939051 PMC6441958

[23] Mason MJ, Zaharakis NM, Sabo R. Reducing Social Stress in Urban Adolescents with Peer Network Counseling. J Child Fam Stud [Internet]. 2016 Dec 1;25(12):3488–96. Available from: doi: 10.1007/s10826-016-0515-5

[24] Killgore WDS, Cloonan SA, Taylor EC, Dailey NS. Loneliness: A signature mental health concern in the era of COVID-19. Psychiatry Res [Internet]. 2020 Aug;290:113117. Available from: doi: 10.1016/j.psychres.2020.113117 32480121 PMC7255345

[25] Kovacs B, Caplan N, Grob S, King M. Social Networks and Loneliness During the COVID-19 Pandemic. Socius [Internet]. 2021 Jan 1;7:2378023120985254. Available from: 10.1177/2378023120985254.

[26] Pang NTP, Tio VCS, Bhupendar Singh AS, Tseu MWL, Shoesmith WD, Abd Rahim MA, et al. Efficacy of a single-session online ACT-based mindfulness intervention among undergraduates in lockdown amid COVID-19 pandemic. Trends Psychiatry Psychother [Internet]. 2021 Aug 11; Available from: doi: 10.47626/2237-6089-2020-0172 34392668 PMC10039730

[27] Offenhauser BR. Can a Self-Compassion Writing Interriting Intervention Impact Fvention Impact Feelings of Loneliness? Available from: https://digitalcommons.macalester.edu/psychology_honors.

[28] Olfson M, Mojtabai R, Sampson NA, Hwang I, Druss B, Wang PS, et al. Dropout from outpatient mental health care in the United States. Psychiatr Serv [Internet]. 2009 Jul;60(7):898–907. Available from: doi: 10.1176/ps.2009.60.7.898 19564219 PMC2774713

[29] Rubin M. Single Session Mindfulness Intervention for Loneliness (SSMILe) Treatment Manual. University of Texas at Austin.

[30] Snow G. blockrand: Randomization for block random clinical trials. R package version 15 [Internet]. 2020; Available from: https://CRAN.R-project.org/package=blockrand.

[31] Hayes AM, Feldman G. Clarifying the construct of mindfulness in the context of emotion regulation and the process of change in therapy. Clin Psychol [Internet]. 2004;11(3):255–62. Available from: http://doi.apa.org/getdoi.cfm?doi=10.1093/clipsy.bph080.

[32] Perlman D, Peplau LA, Goldston SE. Loneliness research: A survey of empirical findings. Preventing the harmful consequences of severe and persistent loneliness [Internet]. 1984;13–46. Available from: https://books.google.com/books?hl=en&lr=&id=x-hEGTrLUOoC&oi=fnd&pg=PA13&dq=defining+loneliness+apa&ots=RLjHkFDb4z&sig=tJ9rShPIYbGTNNMnoUQG6jJCScw.

[33] Hayes SC, Wilson KG, Gifford EV, Follette VM, Strosahl K. Experiential avoidance and behavioral disorders: A functional dimensional approach to diagnosis and treatment [Internet]. Vol. 64, Journal of Consulting and Clinical Psychology. 1996. p. 1152–68. Available from: doi: 10.1037/0022-006x.64.6.1152 8991302

[34] Bishop SR, Lau M, Shapiro S, Carlson L, Anderson ND, Carmody J, et al. Mindfulness: A proposed operational definition. Clinical Psychology: Science and Practice [Internet]. 2004;11(3):230–41. Available from: https://psycnet.apa.org/fulltext/2004-15972-002.pdf.

[35] Baer RA, Smith GT, Lykins E, Button D, Krietemeyer J, Sauer S, et al. Construct validity of the five facet mindfulness questionnaire in meditating and nonmeditating samples. Assessment [Internet]. 2008 Sep;15(3):329–42. Available from: doi: 10.1177/1073191107313003 18310597

[36] Singer T, Klimecki OM. Empathy and compassion. Curr Biol [Internet]. 2014 Sep 22;24(18):R875–8. Available from: doi: 10.1016/j.cub.2014.06.054 25247366

[37] Hays RD, DiMatteo MR. A short-form measure of loneliness. J Pers Assess [Internet]. 1987 Spring;51(1):69–81. Available from: doi: 10.1207/s15327752jpa5101_6 3572711

[38] Cohen S, Kamarck T, Mermelstein R. A global measure of perceived stress. J Health Soc Behav [Internet]. 1983 Dec;24(4):385–96. Available from: https://www.ncbi.nlm.nih.gov/pubmed/6668417. 6668417

[39] Kroenke K, Strine TW, Spitzer RL, Williams JBW, Berry JT, Mokdad AH. The PHQ-8 as a measure of current depression in the general population. J Affect Disord [Internet]. 2009 Apr;114(1–3):163–73. Available from: doi: 10.1016/j.jad.2008.06.026 18752852

[40] Spitzer RL, Kroenke K, Williams JBW, Löwe B. A brief measure for assessing generalized anxiety disorder: the GAD-7. Arch Intern Med [Internet]. 2006 May 22;166(10):1092–7. Available from: doi: 10.1001/archinte.166.10.1092 16717171

[41] Devilly GJ, Borkovec TD. Psychometric properties of the credibility/ expectancy questionnaire. and Experimental Psychiatry. 2000;31:73. 10.1016/s0005-7916(00)00012-4 11132119

[42] Kazdin AE. Therapy outcome questions requiring control of credibility and treatment-generated expectancies. Behav Ther [Internet]. 1979 Jan 1;10(1):81–93. Available from: https://www.sciencedirect.com/science/article/pii/S0005789479800118.

[43] Bürkner PC. Advanced Bayesian multilevel modeling with the R package brms. The R Journal, 10 (1), 395–411. doi: 10.32614/RJ-2018-017; 2018.

