# Supplementary material for: Efficacy of a single session mindfulness based intervention: A randomized clinical trial
Source: PLoS One. 2024 Mar 13;19(3):e0299300. doi: 10.1371/journal.pone.0299300 (PMC10936831; doi:10.1371/journal.pone.0299300)
Supplement: S2 File — (PDF) [file pone.0299300.s002.pdf]

STUDY INFORMATION

For studies following a multi-center or sponsor protocol, please use this guidance to assist in your completion of this form.

For questions regarding definitions, policies, or terms referenced below see the policies and procedures manual.

Study Title and Number from IRBaccess
Preliminary Efficacy of a One-Session Mindfulness Telehealth Intervention for Loneliness
2020-04-0088

Principal Investigator

Name	Position	UT EID	E-mail Address
[redacted]	[redacted]	[redacted]	[redacted]
If principal investigator is a student, describe how the PI is qualified/trained to conduct this study.			
The PI has experience conducting a number of clinical trials supervised by his faculty sponsor [redacted]			

Faculty Sponsor *(required if the PI is a student)*

Name	Position	UT EID	E-mail Address
[redacted]	[redacted]	[redacted]	[redacted]
Describe how faculty sponsor will oversee the conduct of the study.			
[redacted] [redacted] will supervise the study and discuss the study's progress at our weekly lab meetings. If any additional issues arise, individual meetings will be scheduled.			

Primary Point of Contact (if different from PI)

Name	Position	UT EID	E-mail Address

Additional Research Staff

☒	Research staff other than the principal investigator will conduct human subject research.

If additional personnel will be engaged in conducting research human subject research, complete and upload the Research Personnel Form

Engaged in human subject research is defined as contact or interaction with research participants through recruitment, informed consent process, data collection, analysis of or access to identifiable research data.

Purpose and Rationale for Conducting Research
Hypothesis
**Purpose of the Research:** The primary aim of this study is to examine the efficacy of a one-session, hour-long HIPAA-compliant video platform-based mindfulness + compassion telehealth intervention on reducing feelings of loneliness during COVID-19 quarantine.<br><br>**Specific Aims and Hypotheses:**<br>**1: Test the efficacy of a mindfulness + compassion telehealth intervention on reducing symptoms of loneliness.** We predict that participants in the mindfulness + compassion (MC) intervention will show a significantly greater reduction in subjective feelings of loneliness at follow-up relative to those in the mindfulness only (MO) and waitlist control (WL) conditions.<br><br>**2: Examine whether the effects of the intervention on loneliness is mediated by changes in mindfulness.** We predict that changes in mindfulness, as measured by the FFMQ, will mediate the effects of the intervention on feelings of loneliness.<br><br>**3; Examine whether day-to-day practice of mindfulness is causally related to changes in loneliness.** We predict that greater daily practice of mindfulness will be associated with decreases in loneliness day-to-day as measured at the daily assessments.
Study Background
We are currently living in an unprecedented time where individuals are isolated in their homes for an extended period due to the ongoing COVID-19 pandemic. Feelings of loneliness, or perceived social isolation, have often been cited as accompanying objective social isolation (Shankar, McMunn, Banks & Steptoe, 2011). Prior research has established a link between loneliness and a variety of mental health outcomes including increased depressive symptoms (Cacioppo et al., 2006; VanderWeele, Hawkley, Thisted, & Cacioppo, 2011), increased stress reactivity (Thamboo, 2016; Cacioppo et al., 2000), and increased risk of suicidal thoughts and attempts (Conroy & Smith, 1983; Heinrich & Gullone, 2006; Kirkpatrick- Smith, Rich, Bonner, & Jans, 1991; Maris, 1981; Peck, 1983).<br><br>The urgency of the COVID-19 pandemic thus necessitates the investigation of potential short-term interventions for loneliness. Research has suggested that longer-term mindfulness interventions may be effective in mitigating feelings of loneliness and its concomitant mental health outcomes (Zhang et al., 2015; Creswell et al., 2012). The proposed study seeks to determine the preliminary efficacy of a one-session mindfulness-based telehealth intervention for loneliness during COVID-19. To increase the potency of this mindfulness-based intervention, we aim to incorporate compassion motivated social engagement.

There is ample evidence that mindfulness alone is an effective intervention method for addressing loneliness (Zhang et al., 2015; Creswell et al., 2012). However, inclusion of a compassion component may amplify the effects of an intervention on loneliness because prior research has found that less compassion is associated with greater loneliness (Lyon, 2015). To date, no research has explicitly tested the effect of incorporating compassion into a mindfulness intervention for reducing feelings of loneliness.
Design and Methodology
This study will utilize a 3 x 3 randomized controlled trial design with intervention type as a between-subjects factor with three levels (MC, MO, and WL) and time points (baseline, 1-week follow-up, and 2-week follow-up) as a three-level within-subjects factor.
Data Analysis
H1. To address the first hypothesis, we will conduct a mixed effects model with loneliness as the outcome variable, group as the between group variable and assessment as the within group variable, incorporating a random effects structure (random intercept and slope) as indicated by model fitting procedures.<br>H2.To address the second hypothesis we will conduct a mediation analysis with mindfulness at 1-week (controlling for mindfulness at baseline) as the mediator of change in loneliness from baseline to two-week follow-up.<br>H3. To address the third hypothesis, we will use cross-lagged structural equation modeling entering daily loneliness and daily mindfulness practice as the variables for the two weeks of the intervention period.<br><br>All analyses will be conducted using the R statistical software package.

Funding and Regulatory Oversight					
Check all agencies that fund or hold regulatory oversight over the research activities.<br><br>*If study activities are regulated by the FDA, check FDA here. The FDA regulates any experiment that involves a test article and one or more human subjects, and that either must meet the requirements for prior submission to the FDA or the results of which are intended to be later submitted to, or held for inspection by, the FDA as part of an application for a research or marketing permit..*					
☐	Food and Drug Administration (FDA) Regulated				
☐	NIH	☐	Department of Defense (DoD)<br>*Complete Supplemental IRB Application - DoD*	☐	Dept. of Education (DoEd)
☐	Dept. of Energy (DOE)	☐	Department of Justice DOJ/NIJ	☐	Environmental Protection Agency (EPA)
☐	Bureau of Prisons				

☐	Other Federal Agencies:
☐	Industry/Private Sponsor:
	UT Funding Account Number:
☐	Other External Funding:
OSP:	

PROCEDURES

Study Elements					
Check any that apply to your study. This is not meant as a comprehensive record of your entire study.<br><br>*A full description of all study procedures should be provided in the procedures section below or the applicable supplement form.*					
☐	Bio-specimen<br>*Complete Supplemental IRB Application – Biospecimens*	☐	Biometrics	☐	Registry or repository<br>*Complete Supplemental IRB Application - Repository*
☐	Focus Group	☐	Genetic Analysis	☐	Genomic Data Sharing
☐	International research<br>*Complete Supplemental IRB Application - International*	☒	Interview/ Survey	☐	MRI
☐	PHI<br>*Complete Supplemental IRB Application - PHI*	☐	Observation	☐	Record Review (Prospective)
☐	Record Review (Retrospective)	☐	Screening Procedures	☐	Sensors (Externally Placed)
☐	Sensors (Inserted)	☒	Video/Audio Recording	☐	X-Ray
Interventions					
☐	Drug/Biologic<br>*Complete Supplemental IRB Application - Drugs*	☐	Device<br>*Complete Supplemental IRB Application - Device*	☒	Behavioral
Additional Oversight					
☐	Biohazards, Recombinant DNA, or Gene Transfer<br>*Upload IBC approval letter*	☐	Human embryonic, human induced pluripotent, or human totipotent stem cells; or human gametes or embryos	☐	Radiation exposure without direct clinical benefit<br>*Upload radiation safety approval*
Additional Questions:					

☒	This study involves one or more human subjects who are prospectively assigned to one or more interventions (which may include placebo or other control) to evaluate the effects of those interventions on health-related biomedical or behavioral outcomes.

Procedures

Describe all study procedures, including a step-by-step outline of what participants will be asked to do or how data will be used. Be sure to describe all of the following in detail, as applicable:

a) All study procedures, in sequential order
b) All research measures/tests that will be used (state if questions or measures are standardized or published)
c) Secondary data or specimens that will be obtained, how they will be collected, and how they will be used
d) Where each activity will take place, the duration of each, and who will perform each activity
e) Include time commitment of participants
f) Mark all optional procedures as [OPTIONAL]

***All activities will be conducted online via Qualtrics and a HIPAA-compliant video platform.**

Online Screen (20 min.). Interested individuals will complete a web-based screening survey consisting of demographic and COVID-19 related questions and self-report assessments (see measures section).

Eligibility Follow-Up and Consent (10 min.). Individuals determined to be potentially eligible from the online screen will be redirected to a Qualtrics questionnaire containing the informed consent document. After providing consent, participants will be asked to provide their contact information and to sign up for a one-hour intervention time slot with a study clinician (unless in the waitlist control condition, see below).

Randomization. Consenting participants will be randomized to one of three groups: (a) Mindfulness alone; (b) Mindfulness + Compassion; and (c) Wait-list control. Those in the wait-list control condition will be provided with available timeslots following the completion of their one-week follow-up assessment and then randomized to one of the two intervention conditions.

Video Platform-Based Intervention (1 hour).
Participants will be provided a meeting link the day prior to their assigned intervention appointment. See attached for an outline of the MC and MO interventions.

Daily Assessments (5 min.). Participants will complete 7 daily assessments in the week following completion of the intervention. Participants will receive reminder text messages with the daily assessment link via SimpleTexting, a web-based automated messaging software.

Procedures

During these daily assessments, participants will be asked to rate their daily level of sadness, loneliness, anxiety, and stress. Participants will further be asked to complete a mindfulness checklist indicating their practice of mindfulness skills. Last, participants will be asked to report on their daily social interactions. Participants will be provided with reminders and tips regarding mindfulness practice based on their responses.

Booster Sessions (15 min). Participants will be provided with a 15-minute booster session one and two weeks after their intervention session. These booster sessions will allow participants to further hone their mindfulness and/or compassion skills. See attached for a more detailed description of these booster sessions.

Follow-up assessments (20 min). Participants will be sent post-treatment assessments at 1-week and 2-weeks.

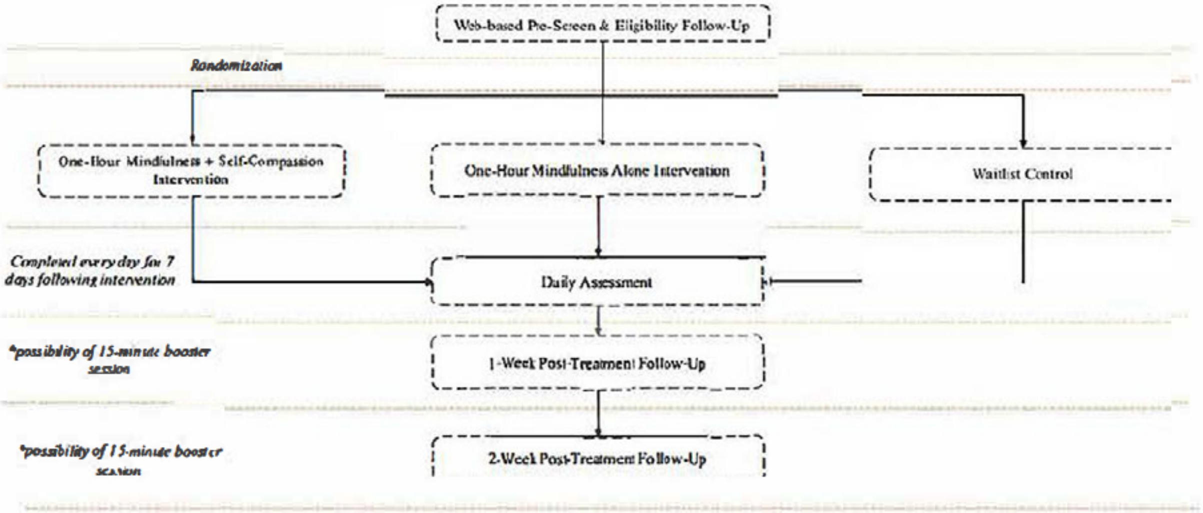

Measures. Self-report assessments will be collected at the web-based pre-screen, one-week post-treatment and two-weeks post-treatment. All assessments will be administered through Qualtrics via a secure internet link.

Demographics and COVID-19 Information. The web-based prescreen will ask participants to provide demographics and COVID-19 information including but not limited to their marital status, age, sex, ethnicity/race, current living arrangements, COVID-19 quarantine and health status, and psychiatric history.

Patient Health Questionnaire – 8 (PHQ-8; Kroenke et al., 2009). The PHQ-8 is an 8-item scale assessing the severity of depressive symptoms. Participants will rate how often they

Procedures

have been bothered by each problem within the last 2 weeks on a 4-point scale from "not at all" to "nearly every day".

Generalized Anxiety Disorder – 7 (GAD-7; Spitzer, Kroenke, Williams, & Lowe, 2006). The GAD-7 is a 7-item self-report measure that assesses the severity of anxiety symptoms. Participants will be asked to rate how often they have been bothered by each symptom in the last 2 weeks on a 4-point scale from "not at all" to "nearly every day".

COVID-19 Perceived Coping Inventory (CPCI; Telch 2020). The CPCI is a 17-item self-report measuring that assesses level of confidence in coping with COVID-19. Participants are asked to rate each item from 0 to 100.

Revised UCLA Loneliness Scale-8 (ULS-8; Hays & DiMatteo, 1987). The ULS-8 is an 8-item self-report measure that assesses subjective feelings of loneliness and social isolation. Participants are asked to rate how often they experience each item on a 4-point scale from "never" to "often". Individual item scores are summed to provide an overall loneliness score.

Five Facet Mindfulness Questionnaire-15 (FFMQ-15; Bear et al., 2007). The FFMQ-15 is a 15-item self-report measures that assesses the five components of mindfulness, including: (1) observation; (2) description; (3) aware actions; (4) non-judgmental inner experience; and (5) non-reactivity. Participants are asked to rate how true each statement is of them on a 5-point scale from "never or very rarely true" to "very often or always true". The FFMQ-15 demonstrates high consistency with scores on alternate versions of the FFMQ (Gu et al., 2016).

Perceived Stress Scale (PSS; Cohen, 1994). The PSS is a 10-item scale that assesses subjective feelings of stress. Participants are asked to rate how often they felt or thought each item on a 5-point scale from "never" to "very often". Individual item scores are summed to provide an overall score.

Santa Clara Brief Compassion Scale (SCBCS; Hwang and Plante, *2008).* The SCBCS is a 5-item self-report measure that assesses compassion. Participants are asked to endorse how often they experience compassion for other on a seven-point scale from "not at all true of me" to "very true of me". Example items include "I tend to feel compassion for people, even though I do not know them."

Interpersonal Support Evaluation List-12 (ISEL-12; Cohen, Mermelstein, Kamarck, & Hoberman, 1985). The ISEL-12 is a 12-item self-report scale that measures the perceived availability of social support on a 4-point scale from "definitely false" to "definitely true". Scores will be summed to provide an overall social support score.

Procedures
***Social Network Index (SNI;* Cohen et al., 1997). The SNI assesses an individual's social network. Participants are asked to report the number of regular social connections they have across domains including family, friends, coworkers, neighbors, etc. Number of people participants see or talk to on a regular basis will be summed to provide a social connectedness score.**<br><br>*Credibility/Expectancy Questionnaire (CEQ)*: The CEQ is a 6 item measure that examines how credible a client thinks a treatment is and their expectations regarding the treatment. The questionnaire demonstrates high internal consistency and test-retest reliability (Devilly & Borkovec, 2000). Participants will only complete this measure at the 1-week follow-up.

Alternatives to Participation in this Study
**Individuals who do not wish to participate in the study will be provided a list of treatment referral resources.**

LOCATIONS

Study Locations

Identify the sites where study activities will occur under the direction of UT Investigators.

☒	UT Austin	☐	UT Health Austin	☐	Dell Seton Medical Center<br>*Upload S.A.T. submission receipt*
☐	Dell Children's Medical Center<br>*Upload S.A.T. submission receipt*	☐	K-12 schools/district	☐	Day care center
☐	Seton Medical Center Austin<br>*Upload S.A.T. submission receipt*	☐	CommunityCare		

External Locations
Include any non-UT site where UT or non-UT personnel will conduct consent, data collection, intervention, or analysis of identifiable data under the direction of the UT principal investigator.

If UT Austin will serve as the reviewing IRB for a multi-site study (study involves collaboration with sites or individuals external to UT Austin who are engaged in human subjects research), contact RSC to verify the UT IRB will serve as the reviewing IRB.<br><br>*Once verified, each relying site must complete the IRB Reliance Form.*

Site Name	IRB Oversight Plan

Additional Questions	
Will UT act as a central coordinating site?	No
Describe procedures to communicate SAEs, UPs, and modifications to external sites.	

SUBJECT POPULATION

Protected Subject Populations					
Select all populations specifically studied under this research.					
☐	Active military personnel	☐	Children	☐	Decisionally impaired adults
☐	Emancipated minors	☐	Fetuses	☐	Individuals with limited English proficiency
☐	Neonates	☐	Pregnant women	☐	Prisoners<br>*Complete Supplemental IRB Application - Repository*
☒	UT Students				

Research Participant Information			
Describe the research population.			
Participant Groups:	• Adults aged 18 – 70		
Age range	18	To	70
Gender	Any		
Inclusion criteria	1. Access to the Internet with teleconferencing for the HIPAA-compliant video platform<br>2. Fluent in English		

	3. Aged 18 – 70 years old<br>4. Currently isolating due to COVID-19<br>5. Endorses loneliness as being among the top three issues impacting their life<br>6. Demonstrates understanding of the constraints of the intervention (e.g. that it is a single session mindfulness training intervention focused on loneliness).<br>7. Has access to a private setting for completing the intervention<br>8. Denies suicidality
Exclusion criteria	1. Trauma as a primary concern<br>2. Significant depression with depression as a primary concern<br>3. Severe mental illness (e.g. bipolar, schizophrenia, borderline personality disorder)
Population info	Adults currently isolating due to COVID-19.

Total Sample Size	
Total number of participants for all participant groups	N = 600
Sample size rationale	600 participants will provide sufficient power for the mediation model accounting for attrition.

SCREENING & RECRUITMENT

Identification and Screening	
☒	This study involves obtaining information or biospecimens for the purpose of screening, recruiting or determining eligibility of prospective subjects prior to informed consent by either:<br>1. Oral or written communication with the prospective subject or LAR<br>2. By accessing records containing identifiable private information or stored identifiable biospecimens

	Describe the identification and/or screening procedures:
	We will utilize a two-stage screening procedure to identify eligible participants. First, interested individuals will complete a 20-minute online screening questionnaire. They will be informed of their preliminary eligibility status upon completion of this survey. Ineligible individuals will be offered access to a list of mental health referral resources. Individuals who meet preliminary eligibility criteria will be redirected to a Qualtrics survey containing the informed consent document.

Recruitment					
Select all recruitment methods utilized for this research and describe the recruitment process. Upload copies of recruitment materials/scripts to IRBaccess.					
☒	E-Mail	☐	Flyer	☐	In-Person
☐	Letter	☒	Social Media	☒	Research Pool
☐	Telephone/Text	☒	Snowball sampling	☒	Web-posting
☒	Word of Mouth	☐	Other:		
Describe the recruitment process including where recruitment will take place.					
Adults living in the United States will be recruited through multiple means, including notices posted on UT Events, announcements on our laboratory website and advertisements on Craigslist and/or social media, as well as word of mouth and through emails to individuals who may be able to forward the materials to individuals in their networks (such as student organizations). These recruitment notices will include a link to our screening questionnaire, which will include a brief overview of the study and its requirements. The specific language used in these posts is included in IRB materials under "Recruitment Materials". The web-screen does not ask interested individuals to include contact information, thereby ensuring personal data will not be obtained via these recruitment means.					

OBTAINING INFORMED CONSENT

Consent Overview			
Select all applicable.<br>*See IRB Policies and Procedures Section 6 for a description of informed consent.*<br>*See IRB Policies and Procedures Section 12.4 for a description of assent/parent permission.*			
☒	Obtaining Written Consent<br>*Complete the Consent and Assent Processes section below*	☐	Requesting Waiver of Documentation of Informed Consent<br>*Complete the Consent and Assent Processes and the Waiver of Documentation of Consent sections below*

☐	Requesting Waiver of Informed Consent<br>*Complete Waiver or Alteration of Informed Consent section below*	☐	Requesting Alteration of the Required Elements of Informed Consent<br>*Complete Waiver or Alteration of Informed Consent section below*
☐	Obtaining Child Assent<br>*Complete the Consent and Assent Processes section below*	☐	Obtaining Short Form Consent<br>*Complete the Consent and Assent Processes section below*

Consent and Assent Processes
Provide a detailed description of the consent process including who will obtain consent, where, and when consent will occur in such a manner that participants have sufficient time for adequate consideration.
Eligible participants will be provided the link to a secure Qualtrics questionnaire containing the ICD. Participants will be able to download an unsigned copy of the ICD on this page. Study personnel contact information will be provided if there are additional questions. A copy of the ICD is attached. Participants will digitally date and sign the ICD and a researcher will co-sign.
Upload consent forms, script, or letter to IRBaccess.

Waiver of Documentation of Consent	
To approve a waiver of documentation of informed consent, one of the following criteria below must be justified by the researcher.<br><br>*Only complete the section below if requesting a waiver of documentation of informed consent.*	
☐ <u>Waiver Option 1</u><br><br>A) The only record linking the subject and the research would be the consent document<br><br>B) The principal risk would be potential harm resulting from a breach of confidentiality.<br><br>C) Each subject will be asked whether the subject wants documentation linking the subject with the research, and the subject's wishes will govern.	 <br><br><br>*Upload consent forms with and without signature lines.*<br><br>*Include this choice in the informed consent form.*<br><br>*Articulate the destruction protocol for signed consent forms in the privacy and confidentiality section.*

☐ **Waiver Option 2**<br><br>**A) This study is minimal risk.**<br><br>**B) Written consent would not be required outside of the research context**	 <br>***Upload consent form to IRBaccess.***
☐ **Waiver Option 3**<br><br>**A) The subjects or legally authorized representatives are members of a distinct cultural group or community in which signing forms is not the norm**<br><br>**B) the research presents no more than minimal risk of harm to subjects.**<br><br>**C) There is an appropriate alternative mechanism for documenting that informed consent was obtained.**	 <br>***Upload consent form to IRBaccess***

Waiver or Alteration of Informed Consent
To approve a waiver of informed consent, all of the following criteria must be justified by the research. Provide a protocol specific justification for each.

Only complete the section below if requesting a waiver of informed consent or alteration of informed consent.		
**The research involves no more than minimal risk to the subjects.**		
**The waiver or alteration will not adversely affect the rights and welfare of the subjects.**		
**The research could not practicably be carried out without the waiver or alteration (it is impracticable to perform the research if obtaining informed consent is required and not just impracticable to obtain consent).**		
**If the research involves using identifiable private information or identifiable biospecimens, the research could not practicably be carried out without using such information or biospecimens in an identifiable format.**		
**Whenever appropriate, the subjects will be provided with additional pertinent information after participation.**	☐	**Additional pertinent information would not be appropriate (*e.g.*, no deception).**
	☐	**Additional pertinent information is appropriate.**
		Research that requires alteration of informed consent on the grounds that deception is necessary must complete the deception section below.
**Deception**		
See IRB Policies and Procedures Section 15 for a description of deception.		
**Describe the nature of deception**	*Click or tap here to enter text.*	

Why is deception required?	[illegible]
Describe debriefing procedures	[illegible]
☐	Research participants will have the opportunity to withdrawal their data during the debriefing.
Upload debriefing form to IRBaccess.	

Consent Translation	
☐	The study population will likely include participants whose limited English speaking status requires translation of the consent form.
The IRB recommends having English versions of consents approved prior to translation.<br>*When available, upload translated documents to IRBaccess.*<br><br>*See IRB Policies and Procedures Section 6.4.1 for a description of translation procedures.*	
☐	The consent documents will be translated by a certified translator.
[illegible]	A non-certified translator will translate the consent documents.
	Describe the translator's qualifications
	[illegible]
	☐ Documents will be translated, and the research team will attest that the translation is accurate and appropriate.
Upload translated documents and attestation (if required) to IRB Access.	

RISKS AND BENEFITS

Benefits		
Compensation for time and effort is not considered a benefit.		
Benefits to Society		Describe scientific and societal benefit.
		The present study may inform whether a brief, web-based mindfulness + compassion intervention is effective in reducing feelings of loneliness during COVID-19 isolation. This intervention could be easily and cost-effectively implemented to help individuals cope with the ongoing crisis.
Direct Benefit	☐	No potential for direct benefits to participants
	☒	Describe potential for direct benefits to participants.
		Participants will learn more about mindfulness and its relationship to loneliness

Risks	
☒	Greater than Minimal Risk Study
	Complete the Data Safety and Monitoring Plan section below.
Research related risks only pertain to risks associated with procedures required by the study; do not include risks of any procedures that the participant would undergo if not participating in the study.	
Describe the risk(s) associated with the research.	
Possible risks associated with this study include: mild to moderate emotional distress in connection with answering survey questions or participating in the intervention.	
Describe the risk mitigation plan	
**Suicidal ideation.** Although unlikely, participants who indicate significant suicidality during the study will be withdrawn, and procedures outlined in our IRB-approved suicidality SOP will be followed (see uploaded SOP).	

Data Safety and Monitoring Boards (DSMBs) and Plans (DSMPs)	
☐	This study will have a DSMB.
	Describe the DSMB including frequency of meetings, members, data reviewed, and stopping points.
☒	The study will have a DSMP.
	Describe the DSMP, including what data or responses are monitored, when data is reviewed, and what actions are taken to react to a safety concern.
	Dr. Telch will be responsible for data and safety monitoring and will provide continuous, close data monitoring. All serious adverse events will be promptly reported to the UT Institutional Review Board (IRB). A report of all non-serious adverse events will be provided to the IRB yearly.

Required Consent Disclosures		
Child and Elder Abuse		
Texas law requires that anyone report suspected child/elder abuse or neglect.		
Is it likely investigators could discover information that would require mandatory reporting by the investigators or staff?	☐	Yes, it is likely. *Include mandated reporting language in applicable informed consent document(s).*
	☒	No, it is not likely.
Incidental Findings		

Incidental findings include: genetic markers, concerning test results, disease, suicidal thoughts, unexpected paternity, engaging in illegal activities.	
☒	It is possible that investigators could discover incidental findings or other information about a participant's previously unknown condition.
	If so, state methods for addressing and reporting incidental findings
	It is possible, but not likely, that a participant may disclose suicidal intent, child or elder abuse during the study which may require reporting to police, or child protective services.
	Include incidental report information as applicable in the informed consent document(s).

Early Withdrawal
List the criteria for withdrawing individual participants from the study (*e.g.*, safety or toxicity concerns, emotional distress, inability to comply with the protocol, or requirements from study sponsor).
Research participants will be withdrawn from the protocol if they report a threat to self or others.
Describe any necessary procedures for ensuring the safety of a participant who has withdrawn early.
Procedures outlined in our IRB-approved suicidality SOP will be followed (see uploaded SOP).
Describe any pre-specified criteria for stopping or changing the study protocol due to safety concerns.
If any of the above are applicable, include this information in your consent form.

PRIVACY AND CONFIDENTIALITY

Privacy
Describe how you will protect the identity and privacy of study participants during each phase of research. Privacy focuses on the individual participants rather than data. In this section, researchers should focus on issues such as where research activities take place and how participant involvement is protected from non-participants.
Include information regarding privacy during identification, recruitment, screening, the consent process, the conduct of the study, and dissemination of data.
All data associated with the project will be kept strictly confidential. Except for the questionnaires that are completed during participant screening, all data will be de-identified with a Qualtrics generated ID number. To further protect subjects' privacy, all data will be paperless with all data being encrypted and stored securely on Qualtrics and UT Box. Only members of the research team will have access to the project's data. Publications resulting from the research will not include any personally identifiable information.

Confidentiality and Data Security Plan

Describe how you will protect the confidentiality of data or address confidentiality concerns.		
☒	Identifiers will be coded to protect confidentiality.	Describe how data is coded and where identifiers are stored.
		We will use Qualtrics - generated user IDs to protect confidentiality. All potential identifying information will be stored separately and securely on UT Qualtrics.
☒	Identifiable data will be destroyed.	Describe destruction plan and timeline
		Identifiable data will be destroyed upon the conclusion of the study.
☐	Identifiable data will not be destroyed.	Provide rationale for retaining identifiable data indefinitely.
Describe how you will store and secure your data (including length, location, and medium of storage):		
All non-identifiable data will be stored in HIPAA secure, encrypted digital files in Qualtrics. Non-identifiable data will be saved indefinitely, whereas files containing identifiable data will be deleted on the date of project completion. Identifiable and non-identifiable information will be stored separately to further protect confidentiality.		

Data Access					
☒	Study team members	☐	Collaborators	☐	Data coordinating center
☐	Sponsor	☒	Future sharing with other researchers	☐	Other: Click or tap here to enter text.
Describe Data Sharing (If Applicable)					
Only non-identifiable data will be shared with other researchers.					

Certificate of Confidentiality		
See IRB Policies and Procedures Section 4.11.5 for a description of a Certificates of Confidentiality.		
☒	The study does not require a Certificate of Confidentiality.	
☐	The study requires a Certificate of Confidentiality.	
	☐	NIH has issued a Certificate of Confidentiality for this study.
	☐	A Certificate of Confidentiality has not been obtained, but there are plans to apply for one.

COMPENSATION AND COSTS

Compensation	
☐	Subjects receive compensation.
☒	Subject will not receive compensation.

Total amount of compensation	[illegible]				
Proration schedule	[illegible]				
When do subjects receive compensation?	[illegible]				
Select the form(s) of compensation					
☐	Cash	☐	Check	☐	Gift Card Click or tap here to enter text.
☐	Course Credit	☐	ClinCard	☐	Other: Click or tap here to enter text.
☐	Compensation amount and type reasonable for this population for the activities requested of them.				
	Click or tap here to enter text.				

Costs			
Select all categories of costs for which participants or their insurance companies will be responsible.			
☒	Participants will have no costs associated with this study		
☐	Standard of care procedures contributing to study data	☐	Research procedures not associated with standard of care
☐	Administration of drugs / devices	☐	Study drugs or devices
☐	Transportation and parking		
☐	[illegible]		

REQUIRED DOCUMENTS

Additional Supporting Documents	
☒	Principal Investigator CV - Required
☒	Faculty Sponsor CV – Required for student PIs
☒	Recruitment Materials
☒	Consent, Parental Permission, and Assent Forms
☒	Measures and Instruments
☐	Sponsor Protocol
☐	Investigator Brochure
☒	Personnel Form
☐	IDE/IND Verification
☐	Supplemental Forms
